# Risk, reward, and suicide: how impulsivity and loss aversion influence decision-making in individuals who have attempted suicide

**DOI:** 10.3389/fpsyt.2026.1791411

**Published:** 2026-04-16

**Authors:** Ani Zerekidze, Lydia Bahlmann, Johannes Petzold, Meng Li, Lejla Colic, Martin Walter, Fabricio Pereira, Mocrane Abbar, Fabrice Jollant, Gerd Wagner

**Affiliations:** 1 Department of Psychiatry and Psychotherapy, Jena University Hospital, Jena, Germany; 2 Department of Psychiatry and Psychotherapy, Carl Gustav Carus University Hospital Dresden, Carl Gustav Carus Faculty of Medicine at Dresden University of Technology, Dresden, Germany; 3 German Center for Mental Health (DZPG), partner site Halle-Jena-Magdeburg, Jena, Germany; 4 Center for Intervention and Research on adaptive and maladaptive brain Circuits underlying mental health (C-I-R-C), Halle-Jena-Magdeburg, Jena, Germany; 5 Department of Clinical Research and Innovation (DRCI), Centre Hospitalier Universitaire (CHU) Nîmes, Nîmes, France; 6 Mathématiques, Informatique, Physique et Application; Département Sciences et Arts, Université de Nîmes, Nîmes, France; 7 Department of psychiatry, Centre Hospitalier Universitaire (CHU) Nîmes, Nîmes, France; 8 Faculty of medicine, University Paris-Saclay, Le Kremlin-Bicetre, France; 9 Department of Psychiatry and addiction, Paul Brousse hospital, APHP, Villejuif, France; 10 Department of psychiatry, McGill Group for Suicide Studies, McGill University, Montreal, QC, Canada

**Keywords:** delay discounting, go/no-go, Iowa gambling task, loss aversion, probability discounting, suicide attempt, value-based decision-making

## Abstract

**Background:**

Previous studies showed impaired decision-making in suicide attempters, but the cognitive mechanisms in play and subgroup differences among attempters need further research. Understanding these differences is crucial for developing targeted interventions.

**Methods:**

For the present case-control study, we recruited 49 depressed patients with histories of both mood disorders and suicide attempts, 34 patient controls with no personal history of suicide attempts, and 49 healthy controls. The participants completed clinical assessments and decision-making tasks: the Iowa-Gambling-Task (IGT), a value-based decision-making battery, a mixed gambling task, and a Go/No-Go task. The study was preregistered at ClinicalTrial.gov (NCT05230043).

**Results:**

Both patient groups showed lower IGT performance, and only suicide attempters lower loss aversion than healthy controls. Compared to both patient and healthy controls, suicide attempters exhibited more total and commission errors on the Go/No-Go task. Subgroup analysis revealed that patients who made an impulsive suicide attempt had higher delay discounting and lower loss aversion rates than healthy controls. Meanwhile, attempters who chose violent means performed worse than those with a non-violent means in the first phase of the IGT and had lower loss aversion compared to both control groups. Finally, poorer IGT performance was associated with lower loss aversion and higher suicidal intent.

**Discussion:**

In addition to deficits in response inhibition in depressed suicide attempters, these findings highlight reduced sensitivity to losses, higher delay discounting and impaired value-based learning in impulsive or violent suicidal acts. They, therefore, underscore the heterogeneity within suicide attempters and highlight the need for individualized approaches in future research and clinical interventions.

## 1 Introduction

Value-based decision-making involves evaluating options based on personal values to maximize expected outcomes, a process integral to daily life (1). The decision to end one’s life can be understood as an extreme outcome of decision-making. In a suicidal crisis, individuals experience a dramatically changing representation of the current problem and the available solutions, often prioritizing immediate relief over long-term consequences (2). This extreme form of decision may stem from impaired cognitive domains such as risk assessment, delayed reward processing, and loss aversion. However, it remains unclear whether these alterations are transient state factors due to a depressive episode, for instance, or long-term trait factors representing elements of sustained vulnerability increasing the risk of repeated attempts.

Growing evidence over the past 25 years suggests that alterations in particular cognitive domains represent vulnerability factors for suicidal behavior (3, 4). Research has notably identified decision-making deficits in suicide attempters (SA), mainly using the Iowa Gambling Task (IGT) (4–8). SA, in particular those who used violent vs non-violent means, made more disadvantageous choices in this task (6, 9, 10). However, the IGT is a complex task, which relies on multiple processes: value-based learning, risk assessment, working memory and cognitive flexibility (11). While previous research has suggested that impaired decision-making in SA is not correlated with deficits in working memory or cognitive control (12), more research is needed to clarify the cognitive mechanisms involved.

Studies using gambling paradigms have suggested that SA may fail to consistently integrate different attributes of an option, such as the magnitude and timing of rewards, into their decisions (13–16). One study demonstrated heightened risk aversion only to probabilistic losses in SA but no differences regarding probabilistic gains compared with depressed patients without previous suicidal behavior and healthy controls (17). Conflicting findings on loss aversion - a tendency to prioritize avoiding losses over acquiring gains (18) - highlight methodological variability. For example, lower loss aversion correlated with higher suicide risk in adolescents (19), yet greater loss aversion was observed in adult SA in a neuroimaging study (17). Such discrepancies may arise from differing operationalizations of loss aversion and sample heterogeneity.

In addition, impulsivity as a multi-dimensional construct plays an important role in decision-making. Heightened impulsivity has been linked to suicidal behavior and may reflect a preference for immediate rewards (e.g., escaping mental pain) over delayed benefits (e.g., problem-solving, positive life experiences) (20, 21). Such a decision can be operationalized with the help of the concept of delay discounting, reflecting the devaluation of delayed rewards (22). A few studies have revealed higher delay discounting rates, i.e. in the older population over 60 years, than in both depressed and healthy older adults (23). However, in a subsequent study, Tsypes, Szanto (24) reported inconsistent valuation of rewards, rather than a true preference for immediate over delayed rewards in older attempters. Delay discounting rate was assessed in both studies using the Monetary Choice Questionnaire. Higher delay discounting rates were also found only among suicide attempters diagnosed with post-traumatic stress disorder (25). Additionally, SA with post-traumatic stress disorder showed a greater influence of immediate rewards on choice behavior (26), suggesting that these effects were driven more by the stress disorder than suicidal behavior.

The inconsistency of the previous results is likely partly due to the heterogeneity of the SA group both in terms of both individual differences and subgroup differences, as in the case of SA who chose a violent suicidal means (27, 28). Some researchers also have argued that premeditated vs impulsive, or high vs low lethality, suicide attempters might represent different phenotypes (14, 29, 30). Subgroup analyses exploring these distinctions are lacking in prior work.

Moreover, only a few studies have investigated the association of response inhibition, another facet of impulsivity, in SA. A suicide attempt history was associated with higher overall errors in the Go/No-Go task (31). In veterans, a higher rate of commission errors—responding incorrectly to non-target stimuli—predicted suicide-related phenomena, such as attempts or ideation, within a 90-day follow-up period (32). Depressed older adults attempters also showed overall worse performance on the Go/No-go task compared to depressed elderly controls (33). In middle-aged SA, elevated number of commission errors was detected relative to control groups in an fMRI study (34). In contrast, Keilp, Gorlyn (35) did not find any differences in commission errors between adult SA and controls.

In the present study, we aimed to i) replicate previously reported impairments in decision-making in SA; ii) address the knowledge gap regarding specific alterations in associated subprocesses and iii) investigate differences according to subgroups of SA (violent; impulsive). In addition to the classical IGT, we adopted a computerized test battery that measures temporal and probability discounting rates based on the Bayesian adaptive approach (36). To overcome some methodological challenges in measuring loss aversion, we adopted a computerized mixed gamble task (37, 38). In addition, we measured response inhibition with the Go-NoGo task, which demonstrates moderate test-retest reliability (39).

Based on previous findings, we hypothesized that SA in comparison to control groups would perform poorly on the IGT; display altered delay discounting and probability discounting rates; make significantly more errors, especially commission errors, in the Go/No-Go task; and exhibit lower loss aversion. We expected SA who used a violent vs. non-violent means to perform significantly worse on the IGT, and SA with an impulsive vs non-impulsive act to show higher delay discounting and commission error rates in the Go/No-Go task.

## 2 Methods

### 2.1 Participants

The present study is part of a larger project entitled SUICIDE_DECIDE, which was conducted from 2021 to 2023 at two different cooperating sites: Jena University Hospital, Germany, and University Hospital of Nimes, France. The study was registered on ClinicalTrial.gov (NCT05230043). It was approved by the following ethic committees: Friedrich Schiller University, Jena, Germany, and the “Comité de Protection des Personnes SUD-EST IV” in France. To meet ethical requirements, prior to the study, all participants gave their informed consent. Individual data were then saved in accordance with the European data protection guidelines (GDRP).

The power estimation for the overall SUICIDE_DECIDE study was based on one of the primary objectives to investigate PFC-striatum structural and functional networks and the understanding of decision-making processes in violent SA. Specifically, it was derived from expected gray matter differences in the caudate between violent and nonviolent SA, based on Jollant, Wagner (40), with an effect size of approximately 1. According to the power calculation, the final target sample size was set to 40 participants per group (20 per site; total N = 160), comprising 40 VSA, 40 nVSA, 40 PC, and 40 HC. This sample size was considered sufficient to detect clinically meaningful group differences while ensuring adequate robustness.

We recruited 132 participants (93 in Jena and 39 in Nîmes), including 49 individuals who met the DSM-5 criteria for both suicidal behavior disorder (i.e., a suicide attempt within the last two years) and major depressive disorder (MDD) (Suicide Attempter, SA). A suicide attempt was defined as “a self-initiated sequence of behaviors by an individual who, at the time of initiation, expected that the set of actions would lead to his or her own death” (41). The time lapse between the last suicide attempt and clinical assessment was on average 31 days (see **Table 1**). We also recruited two control groups: (1) 34 patient controls (PC), of whom 33 had with a current depressive episode of MDD and one with a current depressive episode of bipolar disorder, all with no personal or family history of suicidal behavior in first- or second-degree relatives; (2) 49 healthy controls (HC) with no personal or family history of suicidal behavior or mental disorders in first- or second-degree relatives (**Table 1**).

**Table 1:** Comparison of sociodemographic and clinical characteristics between suicide attempters, patient controls and healthy controls.

Sociodemographic and clinical characteristics	Suicide attempters (SA) (N = 49)	Patient controls (PC) (N = 34)	Healthy controls (HC) (N = 49)	χ^2^/ANOVA *p*	*Post hoc* (Mann-Whitney U)
Age *(Mean ± SD)*	31.3 ± 11.9	33.6 ± 11.6	32.7 ± 9.9	n.s.	
Female *(%)*	67.3	67.6	44.9	= .024	χ^2^: SA, PC>HC*
Education *(%)*				<.001	SA<PC**<HC***
*8 years*	12.2	2.9	2.0		
*10 years*	34.7	14.7	6.1		
*12 years*	53.1	82.4	91.9		
Recurrent MDD *(%)*	55.1	52.9	n.a	n.s.	
Previous depressive episodes (*Mean ± SD)*	4.1 ± 5.3	2.5 ± 3.5	n.a.	n.s.	
MDD/BD onset age in years (*Mean ± SD)*	20.7 ± 10	22.8 ± 9	n.a.	n.s.	
Lifetime N of suicide attempts *(Median, range)*	2 (1-25)	0	0		
Multiple attempts *(%)*	75.5	n.a.	n.a.		
Time since last suicide attempt in days (*Mean ± SD)*	30.8 ± 54.5	n.a.	n.a.		
History of violent suicide attempt(s) in last 12 months *(%)*	44.9%	n.a.	n.a.		
SIS *(last suicide attempt; Mean ± SD)*	18.3 ± 5.3	n.a.	n.a.		
Suicidal Ideation *(item “wish to die” in C-SSRS in last 3 months in %)*	95	50	0		
SB in first degree relatives *(%)*	16.3	0	0		
SB in second degree relatives *(%)*	14.3	0	0		
Current psychiatric medication *(%)*	83.7	70.6	0		
Comorbid diagnosis *(%)*					
*Anxiety disorders*	16.3	11.8	0		
*PTSD*	8.2	0	0		
*OCD*	6.1	0	0		
*Substance use disorder*	36.7	14.7	8.2		
*Eating disorders*	6.1	2.9	0		
MWT-B (IQ)	107.5 ± 14.7	107.6 ± 12.9	105.1 ± 11.9	n.s.	
NART (raw value)	24.6 ± 3.7	24.0 ± 3.50	26.0 ± 3.7	n.s.	
BDI-II	31.8 ± 14	22.1 ± 12.8	2.2 ± 3.6	<.001	SA>PC***>HC***
MADRS	22.8 ± 10	18.6 ± 10.6	1.5 ± 2.2	<.001	SA>PC*>HC***
BHS	12.2 ± 5.2	10.4 ± 4	5.6 ± 3.6	<.001	SA, PC >HC***
ACSS-FAD	19.3 ± 5.1	16.1 ± 5.5	15.5 ± 7.3	= .006	SA>HC**
YMRS	0.9 ± 2	1.1 ± 1.8	0.2 ± 0.7	n.s.	
UPPS					
*Urgency*	30.6 ± 7	28.5 ± 4.9	23.1 ± 5.6	<.001	SA, PC>HC**
*Premeditation*	21.5 ± 5.7	21 ± 4.5	21.1 ± 5.6	n.s.	
*Perseverance*	21.8 ± 6.1	21.4 ± 6.2	17.5 ± 4.7	<.001	SA, PC>HC**
*Sensation seeking*	29.7 ± 8.5	29.1 ± 6.7	29.7 ± 8.5	n.s.	

SD, standard deviation; MDD, major depressive disorder, N, number; SB, Suicide behavior; SIS, Beck’s suicide intent scale; CSSRS, Columbia-Suicide Severity Rating scale; PTSD, post-traumatic stress disorder; OCD, obsessive- compulsive disorder; MWT-B, vocabulary intelligence test; NART, National Adult Reading Test; BDI-II, Beck’s Depression Inventory; MADRS, Montgomery Asberg Depression Scale; ACSS-FAD, Acquired Capability for Suicide Scale-Fearlessness about Death; BHS, Beck’s Hopelessness Scale; YMRS, Young Mania Rating Scale; UPPS, Impulsive Behavior Scale; n.s., non-significant; n.a., not applicable.

* p < 0.05, ** p < 0.01, *** p<0.001.

The inclusion criteria for all participants were being aged between 18 and 60 years and fluent in either German or French. The inclusion criterion for both patient groups was a current depressive episode of MDD or BD. Exclusion criteria were: loss of consciousness due to suicidal act; intellectual disability, assessed using the Multiple Choice Vocabulary Intelligence Test (MWT-B (42);) for the German sample and the National Adult Reading Test (NART) for the French sample (43); current psychotic disorders; current manic, hypomanic, mixed, or rapid cycling episode, severe alcohol or substance use disorder within the last 3 months, as defined by the DSM-5 criteria; electroconvulsive therapy within the last three months; neurological disorders including epilepsy, stroke, traumatic brain injury, and Parkinson’s disease; active inflammatory conditions such as multiple sclerosis and infections; and standard MRI exclusion criteria.

The use of violent suicidal means (N = 22 SA) was classified according to the ICD-10 codes X66-X82. Violent methods include hanging, firearm use, jumping from heights, deep cuts, car crashes, severe burning, gas poisoning, drowning, electrocution, and jumping in front of a train. In contrast, drug overdose and superficial cutting were categorized as non-violent methods.

An impulsive act (N = 20 SA) was defined based on item #15 of the Beck Suicide Intent Sale (SIS). Individuals who reported a premeditation period of up to a maximum of three hours, (corresponding to scores of 0 or 1) were considered to have made an impulsive suicide attempt, reflecting state impulsivity (44, 45).

All participants, regardless of group assignment, were scheduled to complete a standardized three-day assessment sequence. Day 1 consisted of informed consent, verification of inclusion and exclusion criteria, and administration of clinician-rated questionnaires. Day 2 comprised the completion of self-rated questionnaires, neuropsychological assessments and algometry. Day 3 included blood sampling and MRI acquisition.

For their participation in the study, all individuals received 100€. To ensure realistic gambling and increase motivation, all the subjects were informed that they would be paid up to 100€ according to their task performance at the end of the experiments. Importantly, all participants were debriefed immediately after neuropsychological assessment and informed that compensation was fixed at €100 regardless of performance.

### 2.2 Clinical assessment

Trained psychologists with a master’s degree established psychiatric diagnoses using the structured M.I.N.I (46). interview for major psychiatric disorders based on DSM-5 criteria.

Suicidal ideation, suicide intention, and current and past suicide attempt(s) were assessed using the Columbia-Suicide Severity Rating Scale (C-SSRS) (47). Depressive and manic symptoms, as state factors, were evaluated with the Montgomery-Asberg Depression Scale (MADRS) (48), Beck’s Depression Inventory (BDI-II) (49), Beck Hopelessness Scale (BHS) (50) and German and French versions of the Young-Mania-Rating-Scale (YMRS-D) (51, 52).

Impulsivity was assessed using the German and French versions of the UPPS impulsive behavior scale (53), which explores four dimensions of trait impulsivity: lack of premeditation, urgency, sensation seeking, and lack of perseverance.

Finally, the Acquired Capability for Suicide Scale-Fearlessness about Death (ACSS-FAD) was administered to assess the trait of suicide capability (54).

### 2.3 Neuropsychological tasks

To investigate the hypothesized alterations in DM as a potential trait vulnerability factor of SB, the computerized version of the IGT (55, 56) was used (see **Supplementary Materials**). The participants were instructed to choose cards from four decks with the objective of maximizing their winnings. They must learn to avoid risky decks (yielding high gains but higher long-term losses) and choose safer decks (low gains but long-term lower losses). The net score was determined by subtracting the number of disadvantageous from advantageous choices. In addition, five intermediate scores were calculated based on blocks of 20 trials, reflecting the learning rate in their choice patterns throughout the IGT (6, 57, 58).

Delay discounting (DD), probability discounting for gains (PDG) and losses (PDL) were assessed using the computerized VBDM test battery (36). During each trial, participants selected between two options displayed simultaneously on a computer screen, with the chosen option highlighted and used for Bayesian adaptive estimation to generate the next offer near individual indifference points. The discount rate k_o_ was determined by estimating several indifference points at different delays. A strong preference for immediate rewards is indicated by high DD rate. The PDG task involved choices between smaller certain rewards and larger probabilistic rewards, while the PDL task followed a similar procedure. In PDG, high probability discounting rate indicates a preference for certain over probabilistic gains. However, in PDL, higher k values reflect a tendency to select probabilistic over certain losses.

In the mixed gambles task (37), a gamble entailing an equal 50% chance of gaining one amount of money or losing another amount was presented on each of 256 trials. Loss aversion (λ) and the expected value (EV) of the outcome were computed (see **Supplementary Materials**). Subsequent group comparisons were based on λ and β_ev._

To assess response inhibition, we used the computerized Go/No-Go task (59), in which participants were asked to respond to a specific target letter (e.g., “P”) by pressing a key and inhibiting responses to a non-target letter (e.g., “R”). Four parameters were calculated: correct responses to the target (Go) letter, missed responses to the Go letter (omission error), response to non-target (No-Go) letter (commission error) and correct non-response to the No-Go letter. Higher commission errors and smaller reaction times are considered indicators of impulsivity (60).

### 2.4 Statistical analysis

All statistical analyses were conducted using SPSS Statistics version 29 (https://www.ibm.com/de-de/spss). To minimize potential bias during data analysis, statistical analysis was performed by a separate person who was not involved in participant assessment. Furthermore, the dataset provided to the statistician contained numerically coded group labels without any clinical descriptors, effectively ensuring that the statistician was blinded to group membership throughout the analysis.

In the statistical analysis of the mixed gambles task, one participant with more than 20% missing data was excluded. Three subjects in the mixed gambles task and four subjects in the IGT were excluded for non-compliance with task instructions. The outliers in the VBDM were defined as values beyond two standard deviations from the mean. Therefore, five PDG values and seven PDL values were not included in the analysis. To check the normal distribution of the data, the Kolmogorov-Smirnov test was performed. DD (p = 0.2), PDL (p = 0.07), IGT net score (p = 0.12) showed normal distribution, while the Go/No-Go (p < 0.001), EV (p < 0.001) and λ (p < 0.001) and PDG rate (p = 0.037) were non-normally distributed. For normally distributed dependent variables (IGT net score, DD, and PDL rates), analysis of covariance (ANCOVA) was conducted with *group* as the independent variable. *Age, sex*, and *study site* were included as covariates. To correct for multiple comparisons in the *post hoc* tests, the false discovery rate (FDR) method was applied.

For non-normally distributed variables (e.g., the Go/No-Go, PDG and LA parameters), we first conducted a linear regression with *age, sex, and study site*, as independent variables and used the residualized values to test for group differences using the non-parametric Kruskal-Wallis test. For *post hoc* comparisons, the non-parametric Mann-Whitney U test was used, with FDR correction applied for multiple comparisons. In the SA subgroups, residualized values for age, sex, and study site were compared using a t-test or the Mann-Whitney U test. Correlations were calculated using the Spearman’s rank correlation coefficient.

Finally, a stepwise multiple regression was conducted with the IGT net score as the dependent variable and the DD, PDG, PDL rates, Go/No-Go and loss aversion parameters as the independent variables.

## 3 Results

### 3.1 Sociodemographic and clinical measurements

Fewer females and higher education levels were found in the HC as compared to both control groups. However, all the groups displayed similar levels of verbal intelligence. SA reported significantly higher levels of self-reported (BDI-II) and clinician-rated (MADRS) depression severity than PC. SA did not differ significantly from PC in self-rated aspects of impulsivity (UPPS) and hopelessness (BHS), but both patient groups differed significantly from HC in UPPS urgency and UPPS perseverance levels. ACSS-FAD scores were higher in SA than HC.

### 3.2 Neuropsychological tasks

#### 3.2.1 Main group effects

For the IGT, a significant main effect of *group* was found in the ANCOVA (F (2,119) = 3.36, p = 0.013) (**Table 2**). *Post-hoc* comparisons revealed significantly lower net scores in SA (p = 0.01) and PC (p = 0.013) than for HC, with no significant difference between patient groups (p = 0.9). Both comparisons remained significant after FDR correction. When the intermediate scores were analyzed, a significant effect of *group* was observed in the third (F (2,119) = 3.45, p = 0.035), fourth (F (2,119) = 3.21, p = 0.044) and fifth (F (2,119) = 3.12, p = 0.046) 20-trial blocks. Compared with HC, SA had significantly lower net scores in the fourth (p = 0.021) and fifth (p = 0.035) blocks, but did not significantly differ from PC. However, analyses of the intermediate scores did not survive the FDR correction.

**Table 2:** Between group differences in neuropsychological task performances, controlled for sex, age, and site.

Task parameters (Mean ± SD)	Suicide attempters (SA) (N = 49)	Patient controls (PC) (N = 34)	Healthy controls (HC) (N = 49)	*ANCOVA/Kruskal Wallis test* *p*	*Post-Hoc*
Iowa Gambling Task (IGT)					
*Total net score*	1.5 ± 30.9	-0.5 ± 30.6	16.8 ± 29.5	0.013	SA<HC^**^ ***^+^*** PC<HC^*^ ***^+^***
*Net score 1-20^th^ trial*	-4.7± 5.9	-4.4 ± 4.0	-3.9 ± 5.7	n.s.	
*Net score 21-40^th^ trial*	-0.5 ± 7.0	-0.7 ± 6.9	1.9 ± 7.9	n.s.	
*Net score 41-60^th^ trial*	2.2 ± 7.9	0.9 ± 8.4	5.6 ± 9.0	0.035	PC<HC^*^
*Net score 61-80th trial*	1.7 ± 10.8	1.9 ± 9.4	6.5 ± 8.4	0.044	SA<HC^*^
*Net score 81-100^th^ trial*	2.4 ± 10.3	1.7 ± 9.7	6.7 ± 10.1	0.046	SA, PC<HC^*^
VBDM tasks					
*DD, ln (k)*	-5.6 ± 1.9	-5.5 ± 2.1	-5.8 ± 2.0	n.s.	
*PDG, ln (k)*	0.5 ± 1.2	-0.2 ± 1.1	0.4 ± 0.5	0.041	SA>PC^*^ HC>PC^*^
*PDL, ln (k)*	0.00 ± 0.7	0.2 ± 0.8	-0.03 ± 0.6	n.s.	
Go/No-Go					
*Total errors (%)*	26.7 ± 13.8 (8.3%)	22.0 ± 13.1 (6.9%)	20.6 ± 10.2 (6.4%)	0.001	SA>PC^*^ SA>HC^***^ ***^+^***
*Omission errors (%)*	4.8 ± 10.2 (1.5%)	5.4 ± 7.2 (1.7%)	4.0 ± 6.4 (1.3%)	0.049	SA>HC^*^
*Commission errors (%)*	21.9 ± 12.4 (6.8%)	16.6 ± 10.3 (5.2%)	16.7 ± 9.1 (5.2%)	0.011	SA>PC^*^ SA>HC^**^ ***^+^***
Loss aversion task					
*Lambda*	1.1 ± 0.4	1.1 ± 0.2	1.4 ± 1.0	0.039	SA<HC^*^
*EV*	1.6 ± 1.1	1.7 ± 1.2	1.9 ± 1.7	n.s.	

N, number; SD, standard deviation; SA, suicide attempters; PC, patient controls; HC, healthy controls; VBDM, Value-Based Decision-Making battery; DD, Delay Discounting; PDG, Probability Discounting for Gains; PDL, Probability Discounting for Losses; EV, Expected Value. * p < 0.05, ** p < 0.01, **^+^** survives FDR correction.

Regarding VBDM, no significant group effects were found in the ANCOVA for delay discounting (F (2,131) = 1.17, p = 0.3) and for probability discounting for losses (F (2,125) = 1.32, p = 0.3). However, a significant main effect of *group* was detected for probability discounting for gains (H (2) = 6.7, p = 0.041), with PC displaying significantly lower discounting rates compared to both SA (p = 0.037) and HC (p = 0.016) groups (**Table 2**). *Post-hoc* comparisons became non-significant after FDR correction. Overall, the three groups did not differ in reaction times or choice consistency for the three VBDM tasks.

In the Go/No-Go task, the Kruskal-Wallis test revealed significant overall group difference for the total number of errors (H (2) = 13.1, p = 0.001). The number of commission (H (2) = 9.01, p = 0.011) and omission errors (H (2) = 6.0, p = 0.049) differed significantly between groups. SA made significantly more commissions compared to PC (Mann–Whitney U = 478.0, p = 0.033) and HC (Mann–Whitney U = 608.0, p = 0.004). SA also displayed significantly more omission errors than HC (Mann–Whitney U = 668.0, p = 0.019). The *post-hoc* tests for commission errors and total errors in SA versus HC remained significant after FDR correction.

In the loss aversion task, we observed a significant group effect (Kruskal Wallis test, H (2) = 6.48, p = 0.039). In the *post-hoc* comparisons, SA displayed significantly lower loss aversion compared to HC (Mann–Whitney U = 629.0, p = 0.016), but no significant difference was found in comparison to PC (Mann–Whitney U = 497.0, p = 0.07) (**Supplementary Figure 1**). The *post hoc* comparison to HC did not survive FDR correction. Moreover, the expected value did not differ between groups (H (2) = 0.60, p = 0.7).

To further validate the findings, the analysis was repeated after excluding the singe participant with a diagnosis of bipolar disorder. This exclusion did not affect the statistical significance of the primary group effects.

#### 3.2.2 Subgroup analyses

**Figure 1:**
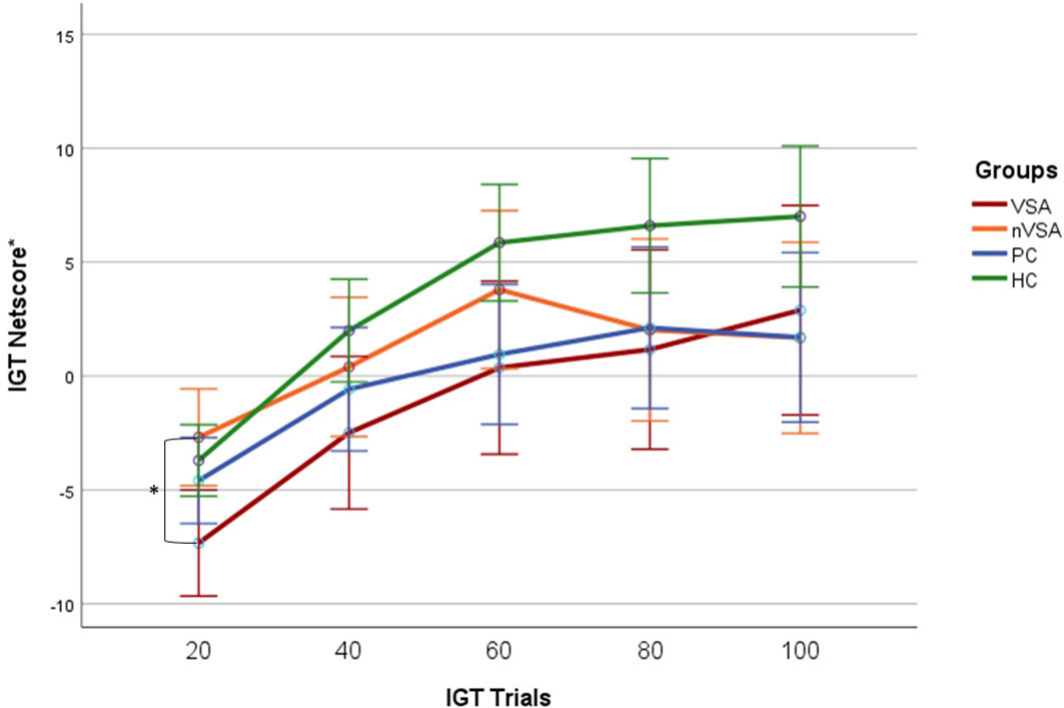
Iowa Gambling Task performance in suicide attempters who used a violent or non-violent suicidal means, and in patient and healthy controls. Participants were instructed to choose cards from four decks with the objective of maximizing their total monetary winnings. Each card selection resulted in either a monetary gain or loss, with a total of 100 decisions made throughout the task. The net score was determined by subtracting the number of disadvantageous choices from the number of advantageous ones. Five intermediate scores were calculated based on blocks of 20 trials, reflecting the learning rate in their choice patterns throughout IGT. IGT, Iowa Gambling Task; VSA, Suicide Attempters who used a violent suicidal means; nVSA, Suicide attempters who used a non-violent suicidal means; PC, Patient Controls; HC, Healthy Controls. * IGT net score residuals, controlled for age, sex, and site.

In the subgroup analyses, we found a significantly lower net score in the first 20 IGT trials among SA who used violent means compared to those who used non-violent suicidal means (Mann–Whitney U = 136.0, p = 0.014). The difference between both subgroups became however non-significant over the course of the task (**Figure 1**). SA used violent suicidal means, but not the ones using non-violent suicidal means, also differed significantly from HC in the first (Mann–Whitney U = 287.0, p = 0.02) and third (Mann–Whitney U = 288.0, p = 0.02) blocks.

**Figure 2:**
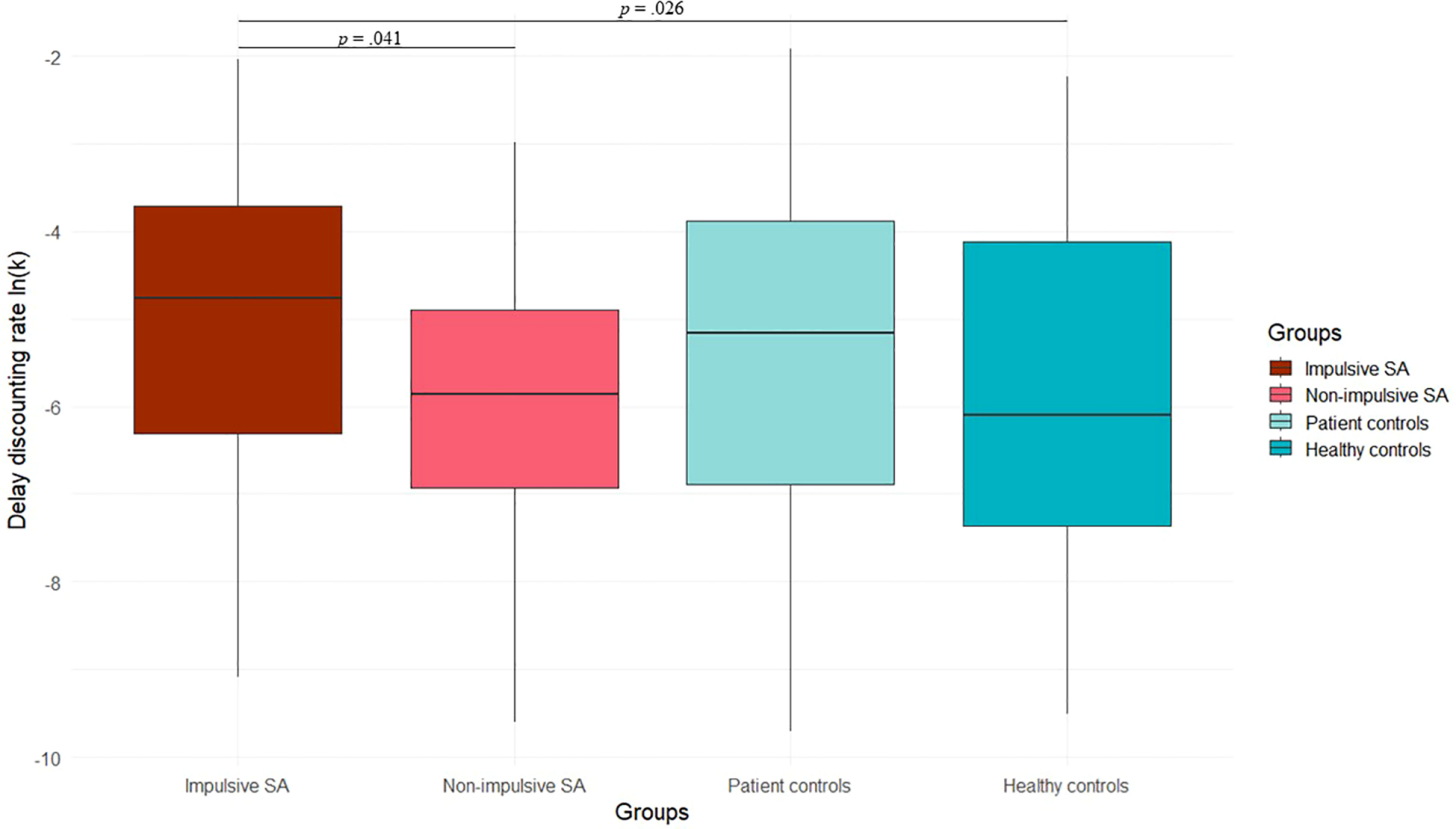
Delay discounting rates in suicide attempters who made an impulsive or non-impulsive suicidal act, and in patient and healthy controls. The discounting rate k_o_ was determined by estimating several indifference points at different delays. To avoid positively skewed parameters, k_o_ was converted using the natural logarithm k = ln(k_o_). SA, Suicide Attempters.

Regarding VBDM tasks, SA with an impulsive act exhibited higher delay discounting rates compared to individuals with a non-impulsive act (t = 1.78, p = 0.041). They also differed significantly from HC in temporal discounting (t = 1.99, p = 0.026; **Figure 2**), but not from PC.

**Figure 3:**
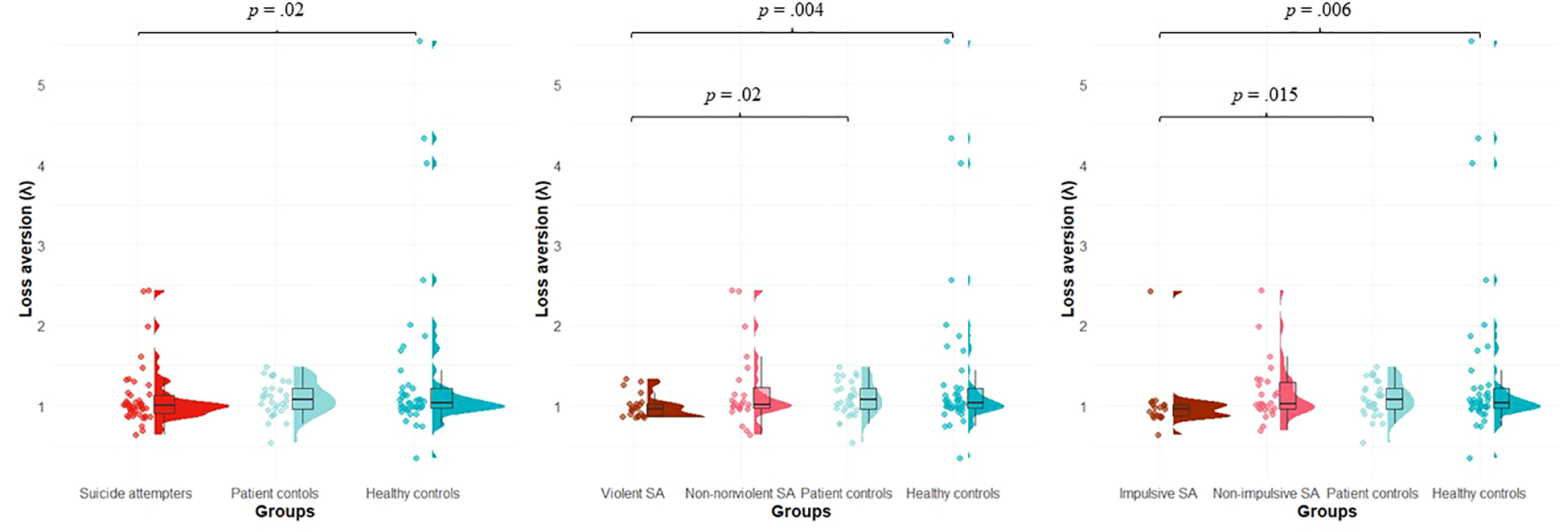
Loss aversion in suicide attempters (total and per subgroups), patient controls and healthy controls. Loss aversion was assessed using the mixed gambles task, comprising 256 trials with a 50% chance of gaining or losing a specific amount of money. Potential gains and losses varied between 5 and 20 Euros. Subjects had to select between four possible responses: strongly accept, weakly accept, weakly reject or strongly reject. A logistic regression in each subject was performed with the amount of potential gains and losses as independent variables and accept/reject categories as dependent variables. Loss aversion (λ) was computed as follows: λ = β_loss_/β_gain_, where β_loss_ and β_gain_ are the unstandardized regression coefficients for the loss and gain variables, respectively. SA, Suicide Attempters.

In the loss aversion task, we did not find any significant differences between SA subgroups. However, when comparing violent suicide attempters to PC (Mann–Whitney U = 204.0, p = 0.02) and HC (Mann–Whitney U = 250.0, p = 0.004), we detected significantly lower loss aversion. SA who used a violent means also showed the lowest dispersion in loss aversion values among the investigated groups. Moreover, SA with an impulsive suicidal act displayed significantly lower loss aversion compared to PC (Mann–Whitney U = 166.0, p = 0.015) and HC (Mann–Whitney U = 216.0, p = 0.006; **Figure 3**).

### 3.3 Correlational analysis in SA

The depression severity was not significantly associated with the IGT total net score and intermediate scores, or DD, PDL and PDG rates. Commission errors (r = 0.33, p = 0.025), and LA (r = 0.308, p = 0.04) significantly correlated with self-reported depression severity in BDI-II. However, these correlations did not survive FDR correction for multiple comparisons.

ACSS total score was significantly associated with commission errors (r = .40, p = .005), which remained significant after adjusting for multiple corrections.

The total number of errors in the Go/No-Go task correlated positively with self-reported impulsivity, i.e., UPPS urgency (r = 0.30, p = 0.04), premeditation (r = 0.29, p = 0.05), perseverance (r = 0.33, p = 0.03) among SA. However, the correlations became non-significant after FDR correction.

### 3.4 Explorative analysis

We conducted a stepwise multiple regression analysis to identify the variables that significantly predict IGT performance in the total sample. The stepwise regression yielded two significant models. The first model (F (1, 98) = 7.24, p = 0.008) included only loss aversion λ (β = 0.264, p = 0.008), which explained about 7% of the variance in IGT performance (R² = 0.069). The second significant (F (1, 98) = 7.24, p = 0.008) model included both parameters of loss aversion, λ (β = 0.307, p = 0.002), and the expected value β_ev_ (β = 0.228, p = 0.021). The predictors explained 12% of the variance in IGT performance (R² = 0.12).

## 4 Discussion

In this study, we examined various aspects of decision-making in SA to identify cognitive alterations that might increase the risk of suicidal behavior. Our study brings novel information about the neuropsychological aspects of suicidal behavior.

We did not fully replicate previous findings regarding IGT performance. While SA made more disadvantageous decisions than HC, they did not differ from PC. Suicide attempters and depressed controls showed comparable neuropsychological performance, indicating that the neuropsychological measures employed in this study may not differentiate suicidal behavior from depression per se. However, it cannot be ruled out that specific cognitive deficits in suicide attempters may be obscured by the overall psychiatric symptom burden, even though no significant correlation between neuropsychological performance and depression severity was detected.

However, in line with our hypothesis, SA who chose a violent suicidal means performed significantly worse than those who chose a non-violent means. These findings are in line with a recent meta-analysis (10). Our findings, therefore, confirm that decision-making impairment is not found in all SA but may be particularly associated with a subgroup of patients who tend to engage in more violent suicidal acts. This population has previously been suggested to differ in terms of sociodemographic, familial, clinical and biological factors; they also showed a higher risk of suicide (27, 40, 61, 62).

IGT deficits were greater in SA using violent means during the early IGT phase, when decisions rely on intuitive emotion-based processes, possibly reflecting lower loss aversion, but not in the late phase. These decisions are thought to be guided by physiological signals (somatic markers) associated with neural activation in the ventromedial prefrontal cortex and amygdala (63). Previous studies have, however, suggested more important deficits during the late phase of the IGT when decisions are based on learning (6). More research will be necessary on the cognitive mechanisms differentiating both subgroups of SA and their role on the choice of the suicidal means.

We also reported lower loss aversion in SA than HC consistent with previous findings (19, 64), with more marked deficits in SA who made an impulsive or violent suicidal act. Regression analysis, furthermore, emphasized the role of loss aversion in IGT performance, with higher loss aversion predicting better net scores. Reduced loss aversion may lead to riskier decision-making, influencing the choice of more violent and lethal means. For individuals with diminished loss aversion, death may not be perceived as a significant loss, lowering the threshold for suicidal behavior and increasing the acquired tendency to attempt for suicide (65).

The neural correlates of loss aversion involve the mesolimbic and mesocortical dopamine systems, which are crucial for reward- and aversion-related cognition (38). Decreased activation in ventral brain regions, including the ventral striatum, ventromedial prefrontal cortex, and medial orbitofrontal cortex, has been observed in response to potential losses (38, 66). IGT performance also involves these neural regions (56, 67). The overlapping neural mechanisms suggest that high loss aversion guides decision-making away from risky choices. Decreased neural activation in these regions has been observed in both SA and first-degree biological relatives of people who died from suicide during risky decision-making tasks (8, 68). Additionally, increased striatal volumes have been found in violent SA (40). These neural changes might underlie the observed impairments in IGT and loss aversion.

One of the pronounced findings was the significantly higher rate of commission errors in the Go/No-Go task among SA compared to both patient and healthy controls. This suggests deficient inhibitory processes, supporting prior studies (32, 34, 69). Poor motor inhibition may represent a trait vulnerability factor facilitating the transition from suicidal ideation to attempts (70), potentially lowering the threshold for suicidal behavior. Furthermore, a higher number of commission errors was associated with greater acquired capability for suicide, reinforcing this perspective. However, contrary to our hypothesis, no significant subgroup differences were observed in the analysis of commission errors.

Our study revealed a correlation between self-reported measures of impulsivity and Go/No-Go performance, that reached significance only at the uncorrected level. Although impulsivity has been linked to suicidal behavior, findings remain inconclusive (20, 71–74). One reason for this is undoubtedly the multidimensional nature of the impulsivity construct (75). The use of different tools for measuring impulsivity likely contributes to inconsistent results (20, 76).

In terms of delay discounting, significant differences were found only among SA who made an impulsive suicidal act compared to those with non-impulsive suicide attempts and HC, providing evidence for the hypothesized higher preference for immediate rewards. Previous research has struggled to link suicidal behavior to trait impulsivity (77), making delay discounting a potentially more specific marker for impulsive suicide attempts. However, a recent study suggests that a suicidal act is more closely linked to inconsistent reward valuation rather than a preference for immediate reward itself (24). This is because SA may struggle to consistently integrate different attributes of an option into their decision-making process. In contrast, the present study did not find any differences in choice consistency between the groups examined. This discrepancy may be due to differences in sample characteristics, such as age, as well as variations in the methods used to assess delay discounting (e.g., questionnaire versus computerized adaptive test).

### 4.1 Limitations

Several limitations should be noted. First, due to recruitment challenges at the French site, fewer participants were recruited than initially planned. Although our study included a relatively large sample, the sample size may still have been insufficient to detect some group differences. However, based on a previous study (8), assuming an effect size (ES) of 1.0 and a statistical power of 90%, a minimum sample size of 18 participants per group would be required to detect group differences using a one-way ANCOVA with three covariates and α = 0.05. For the detection of subgroup differences between violent vs. non-violent SA, assuming the ES of 0.98 reported in the IGT study (78), a sample size of 22 participants per group would be sufficient to detect such differences. Thus, these power analyses suggest that the present study was adequately powered to detect group differences, at least for the IGT. We acknowledge, however, that statistical power for other neuropsychological measures may be more limited.

Second, the ecological validity of the tasks used in this study should be considered when interpreting the results. While gambling tasks model real-life decision-making, they obviously do not fully capture the complexity of decisions like the choice to end one’s life. Nevertheless, the advanced task designs employed likely improve the assessment of underlying neuropsychological constructs.

Third, all patients had a primary diagnosis of a depressive episode and were treated in the hospital for depression. While this ensured diagnostic homogeneity with respect to the primary condition, comorbid psychiatric diagnoses, such as anxiety disorders, or substance use disorders, were present in a subset of participants and were not controlled for in the analyses. Regarding medical comorbidities, rigorous exclusion criteria were applied uniformly across all participants and both study sites: individuals with neurological disorders, active inflammatory conditions, MRI contraindications, or loss of consciousness due to a suicidal act were excluded. While these criteria ensured that major medical confounders were systematically controlled for, the possibility that undetected or unreported medical conditions influenced the results cannot be entirely excluded.

A further potential limitation is the absence of formal blinding of assessors. Given the instruments employed, including the Suicide Intent Scale and C-SSRS, blinding was not feasible, as assessment content inherently reveals participants’ group membership. The use of standardized, structured instruments partially mitigates this limitation by reducing the scope for subjective scorer bias. To further address this potential concern, statistical analyses were performed by a person not involved in participant assessment, with group labels numerically coded without clinical descriptors, ensuring the statistician remained blind to group membership throughout.

Finally, the generalizability of the findings is limited. We recruited middle-aged adults with mood disorders and treated them in university hospitals in Western European countries. Future studies should include SA with other psychiatric conditions, such as borderline personality disorder, to better capture the heterogeneous nature of suicidal behavior. Studies in older adults and adolescents should be conducted more often.

## 5 Conclusions

Our findings reveal impaired response inhibition in SA as well as subgroup differences in decision-making, delay discounting, and loss aversion in relation to the type of suicidal act realized (impulsive or violent). Lower loss aversion may predispose individuals toward riskier decisions, increasing their suicide risk. These results contribute to the ongoing debate surrounding contradictory findings in previous neurocognitive research and underscore the heterogeneity within the SA population. Subgroup analyses are crucial for identifying distinct risk markers and improving suicide prevention efforts by developing new preventative and therapeutic interventions.

## Data Availability

The datasets presented in this article are not readily available because sensitivity of patient data. Requests to access the datasets should be directed to wagner.gerd@uni-jena.de.
