# Supplementary material for: Risk, reward, and suicide: how impulsivity and loss aversion influence decision-making in individuals who have attempted suicide
Source: Front Psychiatry. 2026 Apr 16;17:1791411. doi: 10.3389/fpsyt.2026.1791411 (PMC13130082; doi:10.3389/fpsyt.2026.1791411)
Supplement: Supplementary file 1 [file Supplementaryfile1.docx]

**Title: “Risk, Reward, and Suicide: How Impulsivity and Loss Aversion Influence Decision-Making in Individuals Who Have Attempted Suicide”**

**Authors:** Ani Zerekidze^1^, Lydia Bahlmann^1^, Johannes Petzold^2^, Meng Li^1^, Lejla Colic^1,4^, Martin Walter^1,3,4^, Fabricio Pereira^5^, Mocrane Abbar^6^, Fabrice Jollant^7,8^, Gerd Wagner^1,3*^

*^1^ Department of Psychiatry and Psychotherapy, Jena University Hospital, Jena, Germany.*

*^2^ Department of Psychiatry and Psychotherapy, Carl Gustav Carus University Hospital Dresden, Carl Gustav Carus Faculty of Medicine at Dresden University of Technology, Dresden, Germany.*

*^3^ Center for Intervention and Research on adaptive and maladaptive brain Circuits underlying mental health (C-I-R-C), Halle-Jena-Magdeburg.*

*^4^ German Center for Mental Health (DZPG), partner site Halle-Jena-Magdeburg.*

*^5^ Department of Clinical Research and Innovation (DRCI), CHU Nîmes, Nîmes, France & Mathématiques, Informatique, Physique et Application ; Département Sciences et Arts; Université de Nîmes, Nîmes, France*

*^6^ Department of psychiatry, CHU Nîmes, Nîmes, France*

*^7^ Faculty of medicine, University Paris-Saclay, Le Kremlin-Bicetre, France & Paul Brousse hospital, APHP, Villejuif, France*

*^8^ Department of psychiatry, McGill Group for Suicide Studies, McGill University, Montreal, Canada*

*FJ and GW equally contributed to this work.*

** Corresponding Author: Gerd Wagner, Department of Psychiatry and Psychotherapy, Jena University Hospital, Philosophenweg 3, 07743 Jena, Germany; wagner.gerd@uni-jena.de*

**Supplementary information**

**Neuropsychological tasks**

*Iowa Gambling Task (IGT)*

The computerized version of the IGT (Bechara, Damasio, Damasio, & Lee, 1999; Jollant et al., 2005) was used. Participants were instructed to choose cards from four decks with the objective of maximizing their total monetary winnings. Each card selection resulted in either a monetary gain or loss, with a total of 100 decisions made throughout the task. Two decks (A and B) provided higher gains but also led to even greater losses, making them disadvantageous in the long run. In contrast, the other two decks (C and D) yielded smaller gains but also even fewer losses, resulting in a net gain. Participants were not told the differences between the decks. The choices were categorized as either advantageous (decks C and D) or disadvantageous (decks A and B). The net score was determined by subtracting the number of disadvantageous choices from the number of advantageous ones. In addition, five intermediate scores were calculated based on blocks of 20 trials, reflecting the learning rate in their choice patterns throughout IGT (Bridge et al., 2012; Gorlyn, Keilp, Oquendo, Burke, & John Mann, 2013; Jollant et al., 2005).

*Go/No-Go task*

To assess response inhibition, we adopted the computerized Go/No-Go task (Mueller, 2011), in which participants were asked to respond to a specific target letter (e.g., "P") with pressing a key and inhibit responses to a non-target letter (e.g., "R"). The task was divided into two parts. In the first part of the task, participants had to respond to "P" (Go condition, 80% of trials) and to inhibit responses to "R" (No-Go condition, 20% of trials). In the second part, participants had to respond inversely to "R" (now the Go condition) and to suppress responses to "P" (No-Go condition). In total 320 trials were presented, each condition consisting of 160 trials. Four parameters were calculated: correct responses to the target (Go) letter, missed responses to the Go letter (omission error), response to the non-target (No-Go) letter (commission error) and correct non-response to the No-Go letter. Commission errors and reaction times are considered as indicators of motor impulsivity (Bezdjian, Baker, Lozano, & Raine, 2009).

*Value-based decision-making (VBDM) battery*

Delay discounting (DD), as well as probability discounting for gains (PDG) and losses (PDL) were assessed using the computerized VBDM test battery (Pooseh, Bernhardt, Guevara, Huys, & Smolka, 2018). During each trial, participants selected between two options displayed simultaneously on a computer screen, with the chosen option highlighted and used for Bayesian adaptive estimation to generate the next offer near individual indifference points.

In the DD task, participants indicated preferences between smaller immediate rewards and larger rewards available after varying time delays (e.g., 7€ now or 10€ in 7 days). Delay was set to 3, 7, 14, 31, 61, 180, and 365 days. The discounting rate k_o_ was determined by estimating several indifference points at different delays. To avoid positively skewed parameters, k_o_ was converted using the natural logarithm k = ln(k_o_). Strong preference for immediate rewards is indicated by high DD rate. The PDG task involved choices between smaller certain rewards and larger probabilistic rewards, while the PDL task followed a similar procedure. The probabilities varied between 2/3, 1/2, 1/3, 1/4, and 1/5. In PDG, high probability discounting rate indicates a preference for certain over probabilistic gains. However, in PDL, higher k values reflect a tendency to select probabilistic over sure losses. Each task comprised 50 trials with trials ranging from 3€ to 50€. Choice consistency was additionally assessed by averaging mean-centered ß values across all three tasks, with higher values indicative of consistent preferences across trials.

The tasks were presented on a computer screen using the Psychtoolbox (Brainard, 1997) in MATLAB R2019a (MathWorks Inc., Natick, MA). Before starting the VBDM test battery, all participants were instructed to make their decisions as fast as possible and try to maximize their winnings (PDG) or minimize their losses (PDL). Practice trials were also presented to ensure understanding of each task.

*The mixed gambles task*

In the mixed gambles task (Botvinik-Nezer et al. (2019), a gamble entailing an equal 50% chance of gaining one amount of money or losing another amount was presented on each of 256 trials. Potential gains and losses varied between 5 and 20 Euros (Supplementary figure 1). Subjects had to select between four possible responses: strongly accept, weakly accept, weakly reject or strongly reject. A logistic regression in each subject was performed with the amount of potential gains and losses as independent variables and accept/reject categories as dependent variables. Loss aversion (λ) was computed as follows: λ = β_loss_/β_gain_, where β_loss_ and β_gain_ are the unstandardized regression coefficients for the loss and gain variables, respectively (Tom, Fox, Trepel, & Poldrack, 2007). Additionally, we calculated the expected value of the outcome: EV = 0.5 * LossSum + 0.5 * WinSum. For each subject, EV was used as a dependent variable with the amount of potential gains and losses as independent variables in a logistic regression as well. Subsequent group comparisons were based on λ and β_ev_.

**Supplementary Table 1.** Checklist of items that should be included in reports of case-control studies: STROBE Statement.

|  | | Item No | Recommendation | Paragraph | |
| --- | --- | --- | --- | --- | --- |
| **Title and abstract** | | 1 | (*a*) Indicate the study’s design with a commonly used term in the title or the abstract | Abstract | |
|  |  |  | (*b*) Provide in the abstract an informative and balanced summary of what was done and what was found | Abstract | |
| Introduction | | | | | |
| Background/rationale | | 2 | Explain the scientific background and rationale for the investigation being reported | 1. Introduction | |
| Objectives | | 3 | State specific objectives, including any prespecified hypotheses | 1. Introduction | |
| Methods | | | | | |
| Study design | | 4 | Present key elements of study design early in the paper | 2.1 Participants | |
| Setting | | 5 | Describe the setting, locations, and relevant dates, including periods of recruitment, exposure, follow-up, and data collection | 2.1 Participants | |
| Participants | | 6 | (*a*) Give the eligibility criteria, and the sources and methods of case ascertainment and control selection. Give the rationale for the choice of cases and controls | 2.1 Participants | |
|  |  |  | (*b*) For matched studies, give matching criteria and the number of controls per case | 2.1 Participants | |
| Variables | | 7 | Clearly define all outcomes, exposures, predictors, potential confounders, and effect modifiers. Give diagnostic criteria, if applicable | 2.4 Neuropsychological tasks | |
| Data sources/ measurement | | 8* | For each variable of interest, give sources of data and details of methods of assessment (measurement). Describe comparability of assessment methods if there is more than one group | 2.4 Neuropsychological tasks | |
| Bias | | 9 | Describe any efforts to address potential sources of bias | 2.9 Statistical Analysis | |
| Study size | | 10 | Explain how the study size was arrived at | 2.1 Participants | |
| Quantitative variables | | 11 | Explain how quantitative variables were handled in the analyses. If applicable, describe which groupings were chosen and why | 2.9 Statistical Analysis | |
| Statistical methods | | 12 | (*a*) Describe all statistical methods, including those used to control for confounding | 2.9 Statistical Analysis | |
|  |  |  | (*b*) Describe any methods used to examine subgroups and interactions | 2.9 Statistical Analysis | |
|  |  |  | (*c*) Explain how missing data were addressed | 2.9 Statistical Analysis | |
|  |  |  | (*d*) If applicable, explain how matching of cases and controls was addressed | n.a. | |
|  |  |  | (*e*) Describe any sensitivity analyses | 3.2.1 Main group effects | |
| Results | | | | | |
| Participants | | 13* | (a) Report numbers of individuals at each stage of study—eg numbers potentially eligible, examined for eligibility, confirmed eligible, included in the study, completing follow-up, and analysed | 2.9 Statistical Analysis | |
|  |  |  | (b) Give reasons for non-participation at each stage | 2.9 Statistical Analysis | |
|  |  |  | (c) Consider use of a flow diagram | n.a. | |
| Descriptive data | | 14* | (a) Give characteristics of study participants (eg demographic, clinical, social) and information on exposures and potential confounders | 3.1 Sociodemographic and clinical measurements  Table 1 | |
|  |  |  | (b) Indicate number of participants with missing data for each variable of interest | 2.9 Statistical Analysis | |
| Outcome data | | 15* | Report numbers in each exposure category, or summary measures of exposure | 3.2.1 Main group effects | |
| Main results | | 16 | (*a*) Give unadjusted estimates and, if applicable, confounder-adjusted estimates and their precision (eg, 95% confidence interval). Make clear which confounders were adjusted for and why they were included | 3.2.1 Main group effects |  |
|  |  |  | (*b*) Report category boundaries when continuous variables were categorized | n.a. |  |
|  |  |  | (*c*) If relevant, consider translating estimates of relative risk into absolute risk for a meaningful time period | n.a. |  |
| Other analyses | 17 | Report other analyses done—eg analyses of subgroups and interactions, and sensitivity analyses | | 3.2.2. Subgroup analysis  3.3 Correlational analysis in SA  3.4. Explorative analysis |  |
| Discussion | | | | |  |
| Key results | 18 | Summarise key results with reference to study objectives | | 4. Discussion |  |
| Limitations | 19 | Discuss limitations of the study, taking into account sources of potential bias or imprecision. Discuss both direction and magnitude of any potential bias | | 4.1. Limitations |  |
| Interpretation | 20 | Give a cautious overall interpretation of results considering objectives, limitations, multiplicity of analyses, results from similar studies, and other relevant evidence | | 4. Discussion |  |
| Generalisability | 21 | Discuss the generalisability (external validity) of the study results | | 4.1. Limitations |  |
| Other information | | | | |  |
| Funding | 22 | Give the source of funding and the role of the funders for the present study and, if applicable, for the original study on which the present article is based | | Financial support |  |

**Supplementary figure 1.** Response matrices for all participants. For each participant, the matrix represents the response for each combination of gain (x axis) and loss (y axis) values.


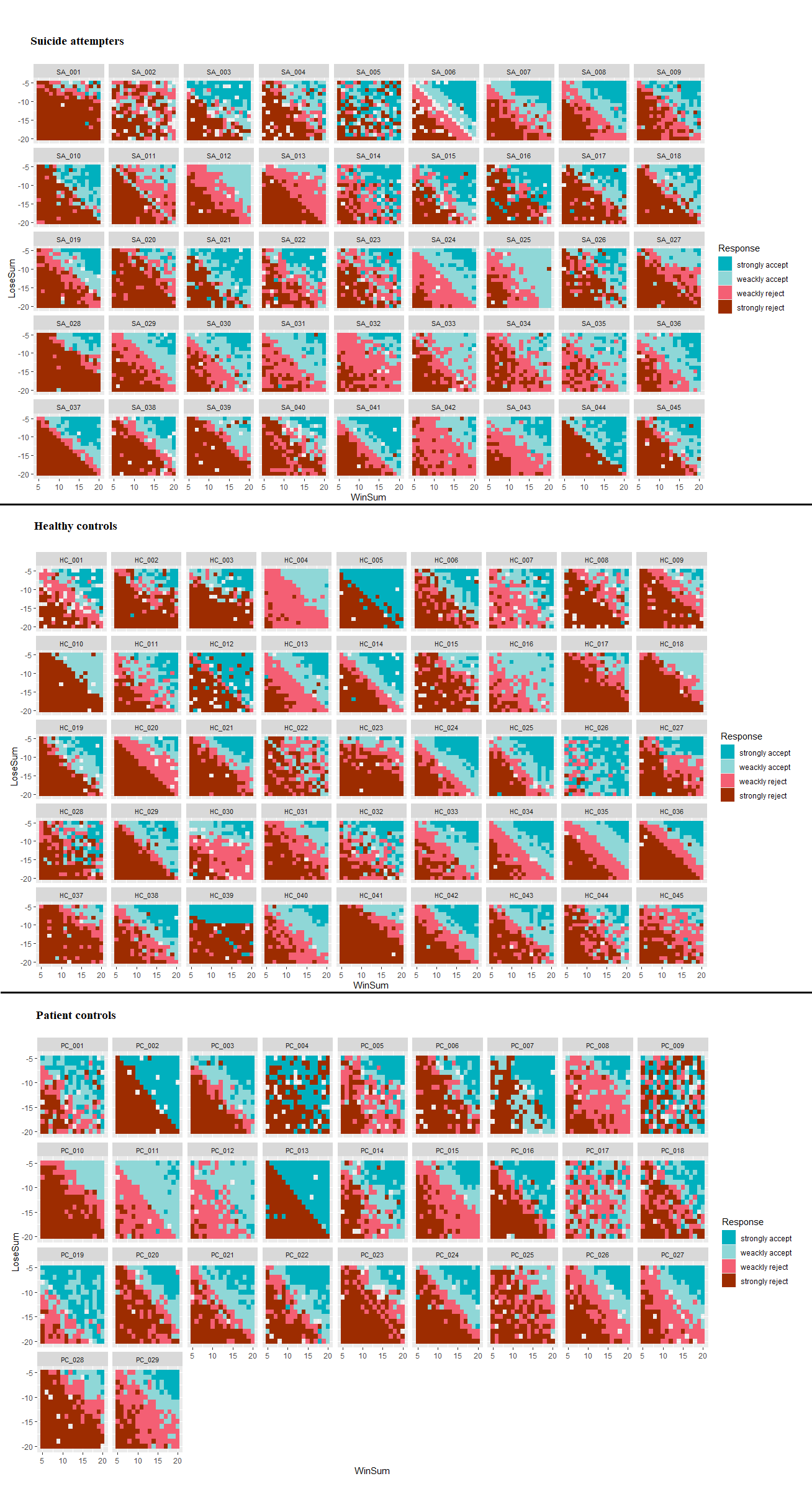


**References:**

Bechara, A., Damasio, H., Damasio, A. R., & Lee, G. P. (1999). Different contributions of the human amygdala and ventromedial prefrontal cortex to decision-making. *Journal of neuroscience, 19*(13), 5473-5481.

Bezdjian, S., Baker, L. A., Lozano, D. I., & Raine, A. (2009). Assessing inattention and impulsivity in children during the Go/NoGo task. *Br J Dev Psychol, 27*(Pt 2), 365-383. doi:10.1348/026151008X314919

Botvinik-Nezer, R., Iwanir, R., Holzmeister, F., Huber, J., Johannesson, M., Kirchler, M., . . . Schonberg, T. (2019). fMRI data of mixed gambles from the Neuroimaging Analysis Replication and Prediction Study. *Sci Data, 6*(1), 106. doi:10.1038/s41597-019-0113-7

Brainard, D. H. (1997). The Psychophysics Toolbox. *Spat Vis, 10*(4), 433-436. Retrieved from <https://www.ncbi.nlm.nih.gov/pubmed/9176952>

Bridge, J. A., McBee-Strayer, S. M., Cannon, E. A., Sheftall, A. H., Reynolds, B., Campo, J. V., . . . Brent, D. A. (2012). Impaired decision making in adolescent suicide attempters. *Journal of the American Academy of Child & Adolescent Psychiatry, 51*(4), 394-403.

Gorlyn, M., Keilp, J. G., Oquendo, M. A., Burke, A. K., & John Mann, J. (2013). Iowa gambling task performance in currently depressed suicide attempters. *Psychiatry Res, 207*(3), 150-157. doi:10.1016/j.psychres.2013.01.030

Jollant, F., Bellivier, F., Leboyer, M., Astruc, B., Torres, S., Verdier, R., . . . Courtet, P. (2005). Impaired decision making in suicide attempters. *Am J Psychiatry, 162*(2), 304-310. doi:162/2/304 [pii]

Mueller, S. (2011). The PEBL Go/No-Go test. *Software recuperado de:* [*http://pebl*](http://pebl)*. sf. net/battery. html*.

Pooseh, S., Bernhardt, N., Guevara, A., Huys, Q. J. M., & Smolka, M. N. (2018). Value-based decision-making battery: A Bayesian adaptive approach to assess impulsive and risky behavior. *Behav Res Methods, 50*(1), 236-249. doi:10.3758/s13428-017-0866-x

Tom, S. M., Fox, C. R., Trepel, C., & Poldrack, R. A. (2007). The neural basis of loss aversion in decision-making under risk. *Science, 315*(5811), 515-518. doi:10.1126/science.1134239
